# Supplementary material for: AIS data-driven MAAC-Stackelberg multi-ship cooperative collision avoidance algorithm
Source: PLoS One. 2026 Jun 3;21(6):e0345950. doi: 10.1371/journal.pone.0345950 (PMC13232843; doi:10.1371/journal.pone.0345950)
Supplement: Attachment — Submitted filename: Response to Reviewers.docx [file pone.0345950.s002.docx]

Response to the Editor and Reviewers

Dear Editor and the two Reviewers,

We sincerely appreciate your meticulous review and valuable comments on our paper entitled "AIS Data-Driven MAAC–Stackelberg Multi-Ship Cooperative Collision Avoidance Algorithm". Your professional suggestions have provided important guidance for improving the quality of the paper. We have systematically revised and improved the paper in response to all the issues raised, and now we would like to present the specific modifications as follows:

Reviewer 1's Comments and Corresponding Revisions

Comment 1

The paper demonstrates good originality and practicality. I recommend publishing after minor revisions.

4.3. Experimental results. Table 3: Performance improvement compared to drl–colregs. It is recommended to add a multi-ship collision AIS trajectory diagram case to visually demonstrate the practical application of the algorithm in Collision Rate Reduction.

Corresponding Revisions

1. We highly appreciate your recognition of the paper's originality and practicality. We have carefully addressed all the issues raised to further improve the paper's quality.

2. In response to the suggestion of adding a multi-ship collision AIS trajectory diagram, we have supplemented Section 4.4 "Visualization of Trajectories and Verification of Safety Performance in Realistic Multi-Ship Encounter Scenarios" in the manuscript. We constructed an 8-ship encounter scenario based on real AIS data from Chengshantou, and added Figure 11 "Visualization of Trajectories" to visually show the collision avoidance trajectory differences between the proposed MAAC-Stackelberg algorithm, DRL-COLREGs, and the Rule-Based Method.

3. In this section, we detailed the key nodes of the trajectory at the 0th, 30th, 60th, and 90th seconds, marked the collision avoidance decision trigger points (red dots), and compared the average minimum safe distance, number of decision points, and trajectory safety status of the three algorithms. The results clearly show that the MAAC-Stackelberg algorithm avoids trajectory crossing and overlapping, and maintains a larger safe distance, which intuitively demonstrates the algorithm's advantage in reducing the collision rate.

4. We have also supplemented the relevant analysis content in the text, explaining the trajectory characteristics and safety performance of each algorithm, so that the experimental results are more intuitive and persuasive.

Reviewer 2 Comments and Corresponding Revisions

Comment Overview

The overall quality of this thesis is acceptable, with clear innovative points that possess certain practical and theoretical reference value. The paper has a complete structure, well-organized sentences, and coherent argumentation logic, being able to support the core argument based on relevant evidence. However, some individual sentences have issues of imprecise expression or slight redundancy, which require further revision and refinement to make the presentation of the full text more concise and rigorous.

Specific Comment 1

There is inconsistent capitalization of core abbreviations in the paper (e.g., "COLREGs" and "AIS" are sometimes written as "colregs" and "ais" without consistent capitalization), and some abbreviations are not fully annotated upon their first occurrence in the main sections. Consistently capitalize all core abbreviations throughout the paper (i.e., AIS, MAAC, COLREGs, BN, LSTM), and clearly present the full name followed by the abbreviation when each is first mentioned, e.g., Automatic Identification System (AIS).

Corresponding Revisions

1. We have standardized the capitalization of all core abbreviations (AIS, MAAC, COLREGs, BN, LSTM) throughout the entire paper, including the abstract, main text, figures, tables, formulas, and references. All lowercase forms such as "ais" and "colregs" have been revised to the correct capitalized versions.

2. For each core abbreviation, we have supplemented the full name annotation when it first appears in the main sections:

- Automatic Identification System (AIS) (first occurrence in Abstract)

- Multi-Agent Actor-Critic (MAAC) (first occurrence in Abstract)

- International Regulations for Preventing Collisions at Sea (COLREGs) (first occurrence in Abstract)

- Bayesian Network (BN) (first occurrence in Abstract)

- Long Short-Term Memory (LSTM) (first occurrence in Abstract)

3. We have checked all abbreviations in the paper to ensure that there is no missing annotation or inconsistent capitalization.

Specific Comment 2

For Fig. 1 (the algorithm framework diagram), only "including the Risk Attitude Perception Layer and the Decision Execution Layer" is described, without clarifying the data flow between the two layers (e.g., how LSTM prediction results are transmitted to BN, and how fused features are input into the MAAC-Stackelberg module). For radar charts, heatmaps, and other figures (Fig. 7–10), only conclusions are provided, with no supplementary interpretation of key data nodes (e.g., the specific value of "Pure MAAC" in the compliance rate dimension in Fig. 7 and the reasons for the differences from the proposed algorithm). Add 1–2 sentences of logical connection and detailed interpretation in the description paragraphs corresponding to these figures.

Corresponding Revisions

1. For Fig. 1 (Algorithm Framework Diagram):

We have supplemented the data flow description between the two layers in the text corresponding to Fig. 1: "In the Risk Attitude Perception Layer, LSTM first predicts the short-term motion states of target ships, and the prediction results are transmitted to BN as key features; BN combines historical AIS data and encounter features to dynamically infer the probabilistic distribution of target ships' risk attitudes. The fused features (including normalized original AIS features, LSTM-predicted motion states, and BN-inferred risk attitude probabilities) are then input into the MAAC-Stackelberg module in the Decision Execution Layer for hierarchical cooperative decision-making."

We have also added data flow arrows and brief labels in Fig. 1 to make the data transmission path more intuitive.

2. For Fig. 7 (Comprehensive Performance Radar Chart):

We have supplemented the interpretation of key data nodes: "In the compliance rate dimension, Pure MAAC only achieves a value of 0.750, which is significantly lower than the 0.920 of the proposed MAAC-Stackelberg algorithm. The reason is that Pure MAAC ignores the rigid constraints of COLREGs and only focuses on collision avoidance goals, leading to failure to strictly implement the starboard steering rule in head-on scenarios and other COLREGs provisions."

3. For Fig. 8 (Overall Performance Score Comparison):

We have added a logical connection sentence: "As shown in Fig. 8, the MAAC-Stackelberg algorithm achieves the highest comprehensive performance score of 0.818 This advantage comes from the balanced performance of the algorithm in multiple dimensions, especially the significant improvement in safety and compliance."

4. For Fig. 9 (3D Spatial Distribution of Comprehensive Performance Scores):

We have supplemented the interpretation: "In the high scenario complexity (≥8) and high environmental uncertainty (≥6) region, the MAAC-Stackelberg algorithm achieves a peak performance score of 0.92, which is 25% higher than the LSTM-only algorithm (0.78). This is because the LSTM-only algorithm lacks a game theory-based hierarchical decision-making mechanism and cannot handle the dynamic switching of leader-follower roles among multiple ships."

5. For Fig. 10 (Performance Advantage Heatmap):

Based on real ship data and simulations, after multiple studies, we have decided to remove this image and related content because it cannot effectively demonstrate the algorithm. As a supplement, we have added a comprehensive table in the discussion, which serves as an overall narrative to reflect the experimental results.

Specific Comment 3

The formatting of the action space expression in Formula (25) is incomplete (missing a closing symbol at the end); the dynamic parameter "a" in Formula (1) is not defined in the context; the units of some variables in the formulas are not specified. 1) Correct the formatting error of Formula (25) by adding the missing closing symbol; 2) Supplement the definition of the parameter "a" in Formula (1) or correct it if it is a typo, and clarify the units of all variables (e.g., unify the unit of Δv_max to "kn"); 3) Add brief explanations for the weight parameters (e.g., α, β) in Formula (8) (Weibull distribution) and Formula (13) (comprehensive risk index) (e.g., "α is a shape parameter reflecting the concentration of collision avoidance behaviors").

Corresponding Revisions

1. Corrected the formatting error of Formula (25): Added the missing closing brace, and the corrected Formula (25)

We have also checked all other formulas in the paper to ensure there are no similar formatting omissions.

2. Supplemented the definition of parameter "a" in Formula (1): After checking, the parameter "a" in Formula (1) is a typo, and the correct parameter is "p" (representing dynamic parameters such as heading ψ and rate of turn r). We have corrected it to "p" and supplemented the definition in the context: "where p represents dynamic parameters such as heading ψ and rate of turn r, μₚ and σₚ are the mean and standard deviation of parameter p in a sliding window with a window size of 30 s, and Δp_max is the maximum allowable change rate of parameter p."

3. Added brief explanations for weight parameters:

- For Formula (8) (Weibull distribution): "α is a shape parameter (without units) reflecting the concentration degree of collision avoidance behavior; when α>1, collision avoidance actions are concentrated (most ships initiate collision avoidance when the TCPA is similar), and when α<1, they are dispersed. β is the scale parameter (unit: s), corresponding to the characteristic value of the collision avoidance action initiation time."

- For Formula (13) (comprehensive risk index): "ωₛ=0.52 is the spatial risk weight, and ωₜ=0.48 is the temporal risk weight, where spatial risk slightly dominates.

Specific Comment 4

The web link in the references does not make sense; it is suggested to delete them.

Corresponding Revisions

1. We have carefully checked the reference list and deleted all invalid web links. For example, we removed the incomplete web link in Reference [17] and ensured that all references comply with the journal's formatting requirements.

2. We have conducted a comprehensive review of the entire reference list to verify the validity and standardization of each citation. For references from ScienceDirect, IEEE Xplore, and other databases, we have standardized the citation format, including the correct DOI number, author names, article titles, journal names, volume, issue, page numbers, and publication year.

3. We have confirmed that the revised reference list is complete, accurate, and in line with the journal's citation specifications.

Specific Comment 5

There are inconsistent expressions of the algorithm name in the paper, such as "maac–Stackelberg", "MAAC-Game–Risk", and "MAAC-Stackelberg"; the hyphenation of "multi-ship" is inconsistent (e.g., "multi-ship", "multi ship", "multiship"). Unify the algorithm name as "MAAC-Stackelberg" (capitalize the initial letters and connect with an English hyphen) and standardize the expression as "multi-ship" throughout the paper.

Corresponding Revisions

1. Unified the algorithm name:

- Standardized all variant names of the algorithm (including "maac–Stackelberg", "MAAC-Game–Risk", etc.) to "MAAC-Stackelberg" throughout the entire manuscript, including the title, abstract, main text, figures, tables, formulas, and references.

- Ensured consistent capitalization (all initial letters of the abbreviation are capitalized) and hyphenation (connected with an English hyphen) for each occurrence of the algorithm name.

2. Standardized the spelling of "multi-ship":

- Corrected all inconsistent spellings (including "multi ship", "multiship") to the unified form "multi-ship" in all contexts, such as scenario descriptions, experimental settings, result analyses, and formula explanations.

- Conducted a full-text search to ensure there are no remaining non-standard expressions.

Specific Comment 6

It is suggested to further clarify the paper’s contribution, polish the language, and eliminate colloquialisms.

We further polished the language of the entire paper and further elaborated on some details.

Eliminated all colloquial expressions and standardized academic wording. For example, revised "can handle" to "can effectively address", "is better than" to "outperforms", and "has a good effect" to "achieves excellent performance".

Optimized sentence logic and structure to enhance the rigor and readability of the paper. For example, split overly long sentences, adjusted the order of clauses to make the argumentation more coherent, and corrected imprecise expressions (e.g., revised "maritime environment" to "maritime navigation environment" for more accuracy).

Checked for redundant content and deleted repetitive descriptions to make the full text more concise.

We would like to express our sincere gratitude again to the editor and the two reviewers for their careful guidance! We have completed all revisions and submitted the revised paper. If further improvements or supplementary materials are needed, we will fully cooperate and respond promptly.

Yours Sincerely

Author: Tie Xu, Tengdong Wang, Jiansen Zhao, Qinyou Hu

Date: 2026.1.25
