# Supplementary material for: AIS data-driven MAAC-Stackelberg multi-ship cooperative collision avoidance algorithm
Source: PLoS One. 2026 Jun 3;21(6):e0345950. doi: 10.1371/journal.pone.0345950 (PMC13232843; doi:10.1371/journal.pone.0345950)
Supplement: Attachment — Submitted filename: Response to Reviewers-2026-3-8.docx [file pone.0345950.s003.docx]

Response to the Reviewers and Editor

Dear Editor and Reviewers,

We sincerely appreciate your careful review and constructive comments on our manuscript entitled "AIS Data-Driven MAAC-Stackelberg Multi-Ship Cooperative Collision Avoidance Algorithm". Your insightful suggestions have been of great value for improving the academic rigor and readability of our work. We have carefully studied all the comments and made comprehensive revisions to the manuscript accordingly. The detailed responses to each comment are presented as follows:

1.Duplicated parameters exist in Table 1. Please revise the description of related parameters and use alternative notations for relative orientation symbols to avoid confusion with other symbols throughout the manuscript.
 We have carefully checked and revised Table 1 by removing duplicated parameters and standardizing the description of all related parameters. Meanwhile,we have adopted new alternative notations for the relative orientation symbols and verified the consistency of all symbols across the full manuscript to eliminate ambiguity and confusion.
2. In Eq. (32) ,the selection criterion of the exploration noise coefficient is not specified. Additionally, please add a schematic diagram of the algorithm for better illustration.
 We have supplemented the detailed selection criterion and theoretical basis of the exploration noise coefficient corresponding to Eq. (32) in the manuscript. Furthermore, we have added a clear schematic diagram of the proposed algorithm to visually demonstrate its framework and workflow, which helps readers better understand the mechanism of the algorithm.
3. The presentation in Section 4.4.1 (statistical significance test) should be further improved; the comparison of advantages among different algorithms in Section 4.3.3 needs to be more concise.
 We have optimized the presentation and logical organization of the statistical significance test in Section 4.4.1 to enhance its readability and rigor. For the comparison of algorithmic advantages in Section 4.3.3, we have streamlined the content, highlighted the core strengths and key differences, and removed redundant descriptions to make the comparison more focused and concise.
4. The overall language and writing style of the manuscript should be further polished

We have conducted a thorough polishing of the entire manuscript, including grammar refinement, sentence structure optimization, and unification of academic expressions. The language has been revised to be more accurate, fluent, and consistent with standard academic writing norms.

We sincerely hope that the revised manuscript can meet the requirements of the journal. Thank you again for your professional and constructive suggestions.

Best regards,

Author: Tie Xu, Tengdong Wang, Jiansen Zhao, Qinyou Hu

Date: 2026.3.8
